# The influence of friction on the determination of rock fracture toughness

**DOI:** 10.1038/s41598-022-11046-6

**Published:** 2022-05-05

**Authors:** Qin Xie, Yuan Zeng, Shengxiang Li, Xiling Liu, Kun Du

**Affiliations:** School of Resources and Safety Engineering, Central South University, Changsha, 410083 Hunan China

**Keywords:** Civil engineering, Materials science, Solid Earth sciences

## Abstract

The accurate determination of fracture toughness (*K*_Ic_) in rock is of great significance to the study of rock fracture characteristics. However, the effect of contact friction in the loading process on the test results of fracture toughness is rarely considered, and the tested *K*_Ic_ in previous studies is generally higher than the inherent *K*_Ic_ of the material. Based on the ABAQUS software, the influence of friction on the test results of *K*_Ic_ was investigated under different material elastic moduli, loading conditions and specimen sizes by the finite element analysis in this study. The results show that for the specimen with the notch semi-circular bend configuration, if the presence of friction is considered, the changes of load level, the span of roller support and the specimen size can significantly affect the test results of fracture toughness, except the elastic modulus of the material; if the influence of friction is not considered, there will be a large deviation in the research results of the size effect of fracture toughness in rock. As the friction coefficient increases, the estimated value of the fracture energy increases, while the fracture process zone length decreases for the specimen with an infinite size.

## Introduction

The process of earthquake preparation and occurrence is closely related to the crack propagation in the rock mass. To comprehensively explore the mechanism of earthquake preparation, the propagation of prefabricated cracks in rock can be studied to simulate the propagation of the existing faults subjected to the subsequent regional stress field. The fracture toughness (*K*_Ic_) of the rock mass is a representative parameter to measure the resistance of rock materials to crack propagation. During an earthquake, when the stress intensity factor (*K*_I_) of the rock mass is greater than the *K*_Ic_ of the rock mass, the crack will expand unsteadily until a relatively hard and thick rock mass is encountered. Therefore, the accurate determination of *K*_Ic_ is significant to the research on earthquake preparation and occurrence.

Based on the stress analysis and crack propagation path, cracks can be divided into three types, namely open cracks (mode I), sliding cracks (mode II) and tearing cracks (mode III). Among the three cracks, mode I cracks are the most common and most dangerous ones, and the mode I fracture toughness is tested in more varied ways than the other modes. The International Society of Rock Mechanics (ISRM) recommends four test methods for the standard mode I fracture toughness, including the cracked chevron notched Brazilian disc (CCNBD) configuration^1,2^, short rod (SR) configuration^3^, the chevron bend (CB) configuration^3^ and the notch semi-circular bend (NSCB) configuration^4^. In addition, some non-standard fracture toughness test methods have also been widely used, such as the straight notch disk bend (SNDB)^5,6^, the chevron notch semi-circular bend (CNSCB)^7,8^ and the cracked straight through Brazilian disc (CSTBD)^9–11^. However, there are sometimes great differences in the *K*_Ic_ measured by the same rock material with different configurations^12–15^. Aliha et al.^16^ found that the mode I fracture resistance is significantly dependent on the geometry and loading conditions of the test specimen, and believed that the effect of the higher-order stress term is the root cause for the difference in fracture resistance of specimens. On this basis, the subsequent research on the prediction of mode I and mode II fracture toughness of specimens with different configurations has been carried out^17–26^. It is worth noting that the latest studies have found that the friction between the loading equipment and the specimen surface can also significantly affect the measurement results of *K*_Ic_. Sedighi et al.^27^ reported that for the rock specimen with the NSCB configuration, the change of friction coefficient between roller support and specimen surface may increase the tested *K*_Ic_ about twice. By establishing the finite element numerical model, Wu et al.^28^ claimed that different from specimens with the NSCB configuration, the effect of the friction between the loaded plate and the specimen surface on the *K*_I_ of the rock specimen with the CSTBD configuration can be ignored. Due to the advantages of convenient processing and operation, NSCB configuration has been widely used in quasi-brittle materials (such as rock and concrete) to determine the *K*_Ic_^29–38^. Ghouli et al.^30^ established the size effect law of the *K*_Ic_ by using specimens with NSCB configuration. Xu et al.^31^ determined the relationship between rock initiation and instability fracture toughness and specimen size by specimens with the NSCB configuration. Pakdaman et al.^38^ analyzed the difference of the *K*_Ic_ determined by specimens with different configurations including NSCB configuration via numerical and experimental methods. However, the friction between the specimen surface and the roller support was not considered in specimens with the NSCB configuration in the above studies. Although Sedighi et al.^27^ believed that the effect of friction on the test of the *K*_Ic_ is the same at any load level, the friction force experienced by the specimen under different load levels is significantly different. Besides, previous studies only studied the influence of friction on the *K*_I_ (the first term of Williams’ stress expansion), but did not study the influence of friction on the higher-order term of Williams’ expansion^39^. The influence of friction on the establishment of the size effect law of fracture toughness of rock specimens has also never been investigated.

In this study, the influence of different friction forces on the *K*_I_ of the specimen was analyzed. Meanwhile, the effects of friction on *K*_I_ and tensile stress at the crack tip front of the specimen were compared under different elastic modulus, load level and roller support span. Finally, based on the variation law of *K*_Ic_ with the specimen size obtained in previous studies, the influence of friction on the establishment of the Bazant's size effect law, the fracture energy and the fracture process zone length for the specimen with an infinite size were considered. In addition, the variations of fracture process zone length with the specimen size under different friction coefficients were also analyzed.

## Methods

**Figure 1:**
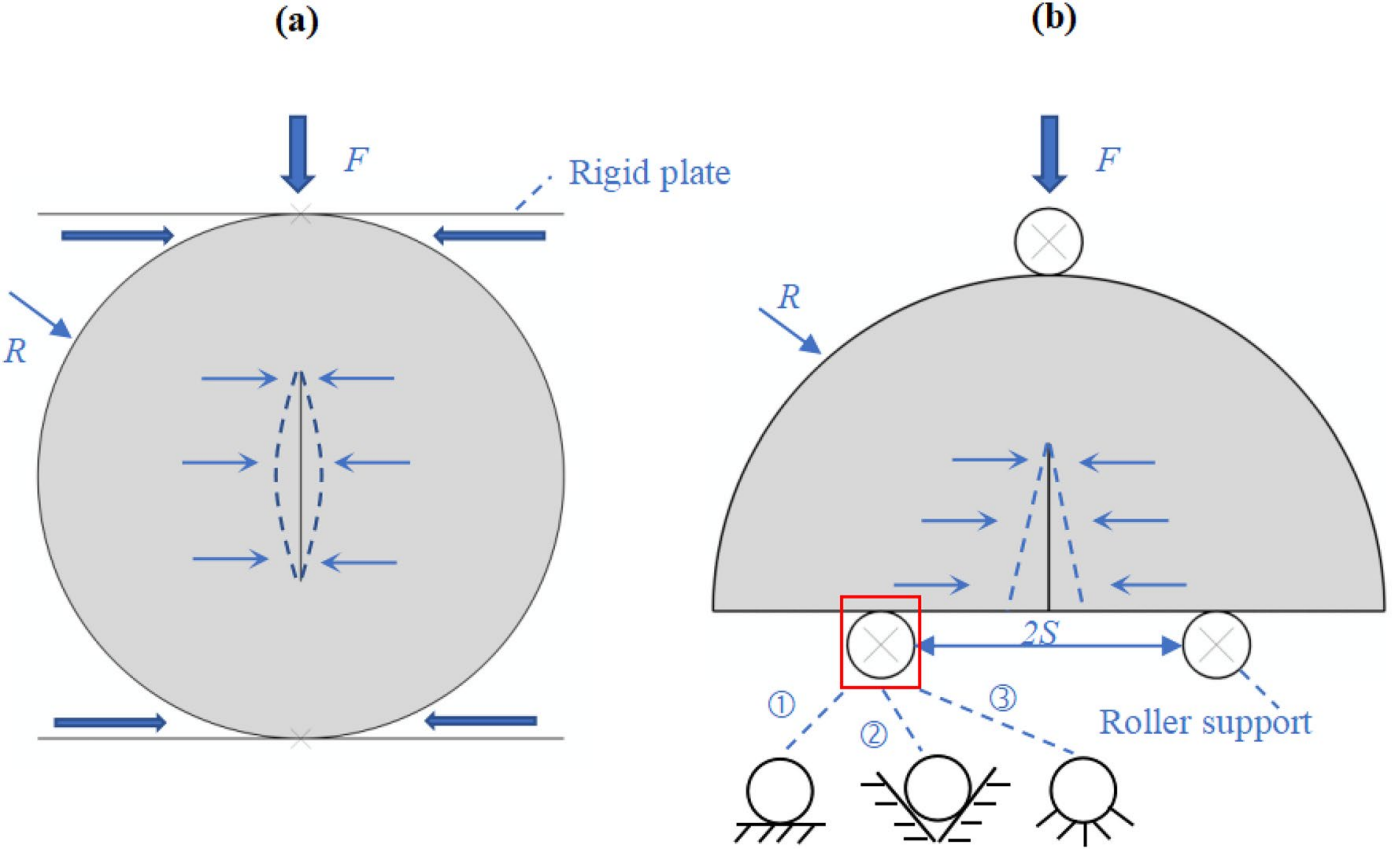
Loading diagram of rock specimens with (**a**) CSTBD configuration and (**b**) NSCB configuration. The three common placement methods of roller support for NSCB configuration: ① free placement; ② put it into the V-shaped groove; ③ fixed.

In the previous studies, the difference between full disk and half-disk specimens in fracture toughness tests have been compared^40–42^**.** Rock specimens with the CSTBD configuration and NSCB configuration were selected in this study, and the influence of contact friction on *K*_I_ was analyzed. Figure 1 shows the loading diagram of rock specimens with CSTBD configuration and NSCB configuration. For rock specimens with the CSTBD configuration, due to the Poisson effect, the tensile stress in the transverse direction can be produced by the compression deformation of the specimen in the longitudinal direction, leading to the failure of the specimen eventually. For rock specimens with the NSCB configuration, the load is applied on the top of the specimen parallel to the crack direction, and two roller supports are symmetrically arranged on both sides of the crack, and the specimen will fail under the action of a tensile moment. It should be noted that when the specimen surface is not smooth, friction effect does exist in the two test methods of *K*_Ic_. Generally, friction can be divided into sliding friction and rolling friction. For the loading process of rock specimens with the CSTBD configuration, sliding friction only exists since the loading plate cannot roll. For rock specimens with the NSCB configurations, the form of friction depends on the placement of the roller support. Figure 1b shows three common placement methods of roller support. The first method is free placement, and the friction on the contact surface belongs to rolling friction because the roller support can roll. The second method is to put the roller support into the V-shaped groove. In this way, the roller support can still roll, but the rolling friction is greater than that of free placement. The third method is the fixed roller support, and the friction on the contact surface becomes sliding friction. Obviously, the sliding friction force is much greater than the rolling friction force. Therefore, the friction of the fixed roller support on the specimens is much greater than that of the first two placement methods. In order to facilitate the analysis, we directly change the friction coefficient in the numerical model to represent the friction force on the specimen under various loading conditions.

**Figure 2:**
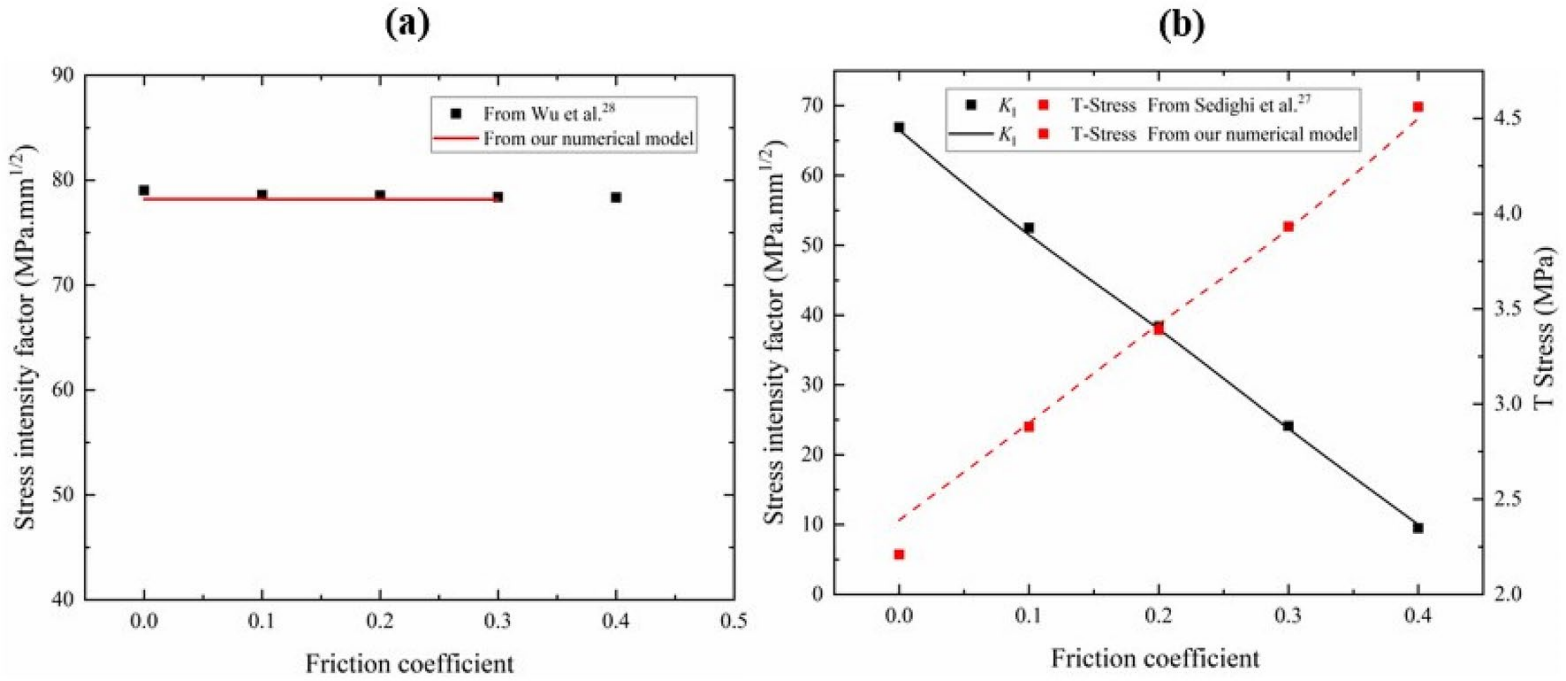
The trends for stress intensity factor of rock specimens with (**a**) CSTBD configuration and (**b**) NSCB configuration against the friction coefficient.

**Figure 3:**
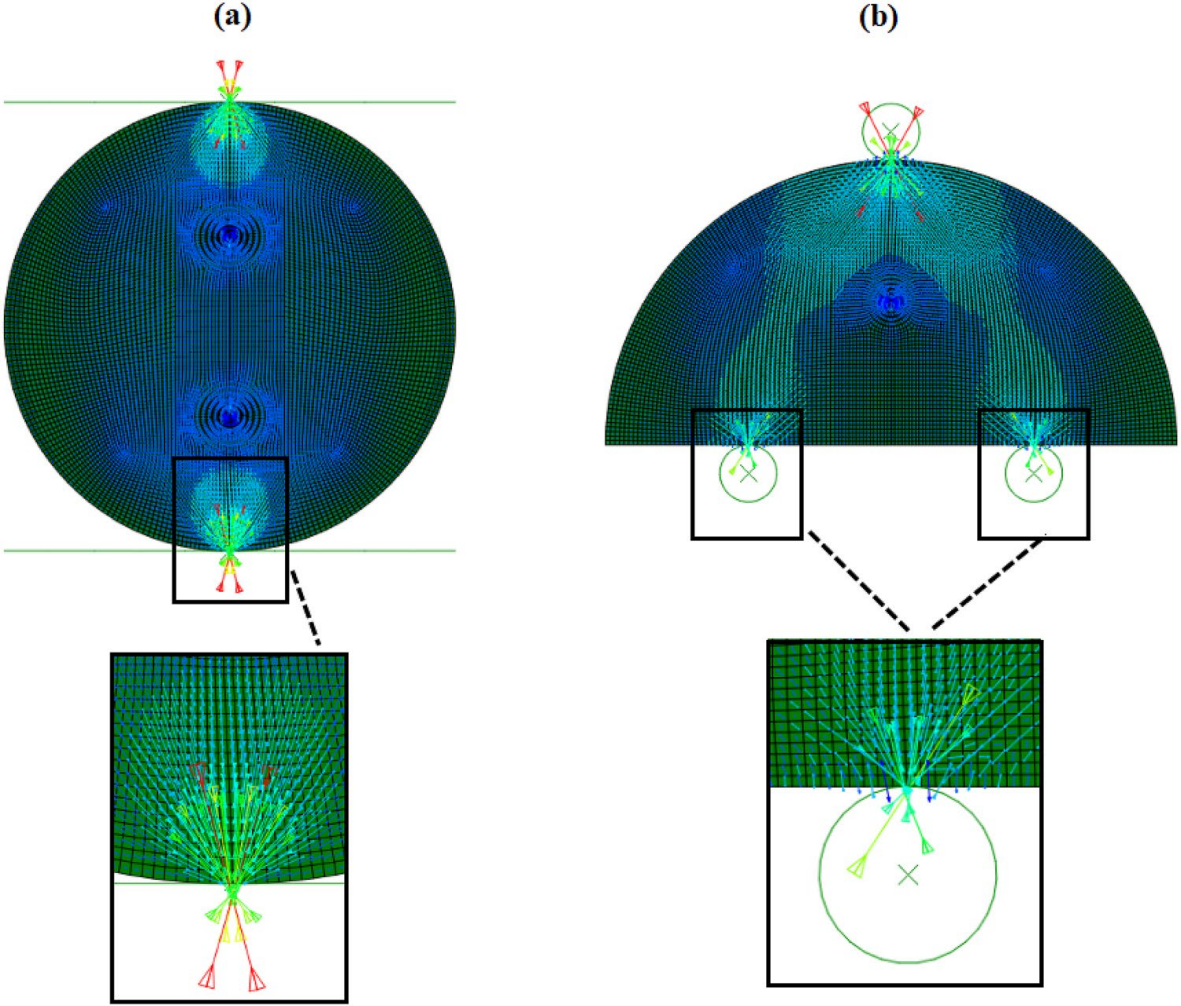
The orientation of principal stresses rock specimens with of (**a**) CSTBD configuration and (**b**) NSCB configuration determined by finite element method at friction coefficient of 0.2. The figure is exported from ABAQUS software 2016.

To verify the output results of the numerical model established in this study, we first reproduce the results of a qualified numerical model in previous studies^27,28^. The numerical models of rock specimens with CSTBD configuration and NSCB configuration established in ABAQUS software. ABAQUS software has been widely used to solve the stress intensity factors of models under various complex conditions, and the simulation results of this software are consistent with that obtained by theoretical methods^43–50^. Table 1 shows the material parameters, load level and specimen size in the model. The corresponding loads and boundary conditions are applied to the numerical model, and the advanced algorithms are used in the grid division module. As shown in Fig. 2, the *K*_I_ of rock specimens with the CSTBD configuration and the NSCB configuration change with the change of the friction coefficient. For rock specimens with CSTBD configuration, the *K*_I_ does not change significantly with the increase of friction coefficient, indicating that the effect of friction in the CSTBD configuration on test results of *K*_Ic_ can be ignored. This finding is consistent with the results reported in previous studies^28^. For rock specimens with NSCB configuration, the *K*_I_ decreases significantly with the increase of friction coefficient. It indicates that when rock specimens with NSCB configuration are used to test *K*_Ic_, the placement of roller support should be considered, and the friction coefficient should be minimized to obtain a reasonable test value of *K*_Ic_. In addition, with the change of friction coefficient, the variation of the *K*_I_ of rock specimens with the NSCB configuration is in good agreement with that reported by Sedighi et al.^27^, which further verifies the reliability of the proposed numerical model established in this study. Figure 3 shows the maximum principal stress direction of rock specimens with the CSTBD configuration and NSCB configuration at the friction coefficient of 0.2. Compared to specimens with the CSTBD configuration, the maximum principal stress distribution at the contact between the specimen with NSCB configuration and the roller support is asymmetric. This is because the additional friction disrupts the original symmetrical stress distribution. It can be seen that there is friction in the specimen with the NSCB configuration, while there is almost no friction at the contact in the specimen with the CSTBD configuration.

**Table 1:** Input parameters used in rock models with CSTBD and NSCB configurations.

Description	CSTBD configuration	NSCB configuration
Specimen radius, *R* (mm)	31.5	50
Notch length, *a* (mm)	25.2	25
Half the span of roller support, *S* (mm)	–	25
Friction coefficients between the rigid plate or Roller support and the specimen	0,0.1,0.2,0.3,0.4	0,0.1,0.2,0.3,0.4
Element type	CPS4R, CPS3	
Elastic modulus, *E* (GPa)	26.77	2.9
Poisson’s ratio	0.2	0.38
Load, *F* (N)	1000	1000

## Results and discussion

### Effect of elastic modulus of rock on the fracture toughness test

Since the NSCB configuration recommended by ISRM for testing *K*_Ic_ has no explicit requirement for material properties of rock, the NSCB configuration can be applied to any rock materials by default. Besides, the existence of friction between the specimen surface and the roller support is not considered in the recommendation. Therefore, it is necessary to discuss the influence of the change of material properties on the fracture toughness test. In this study, four kinds of rock materials with large differences in elastic modulus are selected for analysis. Table 2 shows the basic material parameters of the four rocks and their values in the numerical model. Since the difference in Poisson's ratio of the four rock materials is not significant, the influence of Poisson's ratio on the fracture toughness test of the rock mass is not considered in the presence of friction.

**Table 2:** Basic material properties of four rocks and input parameters in the model.

Rock type	*E* (GPa)		Poisson's ratio	
	Range	In model	Range	In model
Diorite	101.0–117.5	110	0.26–0.37	0.3
Granite	54.9–57.5	55	0.13–0.23	
Sandstone	27.9–47.6	30	0.15–0.52	
Gneiss	14.0–55.1	15	0.2–0.34	

**Figure 4:**
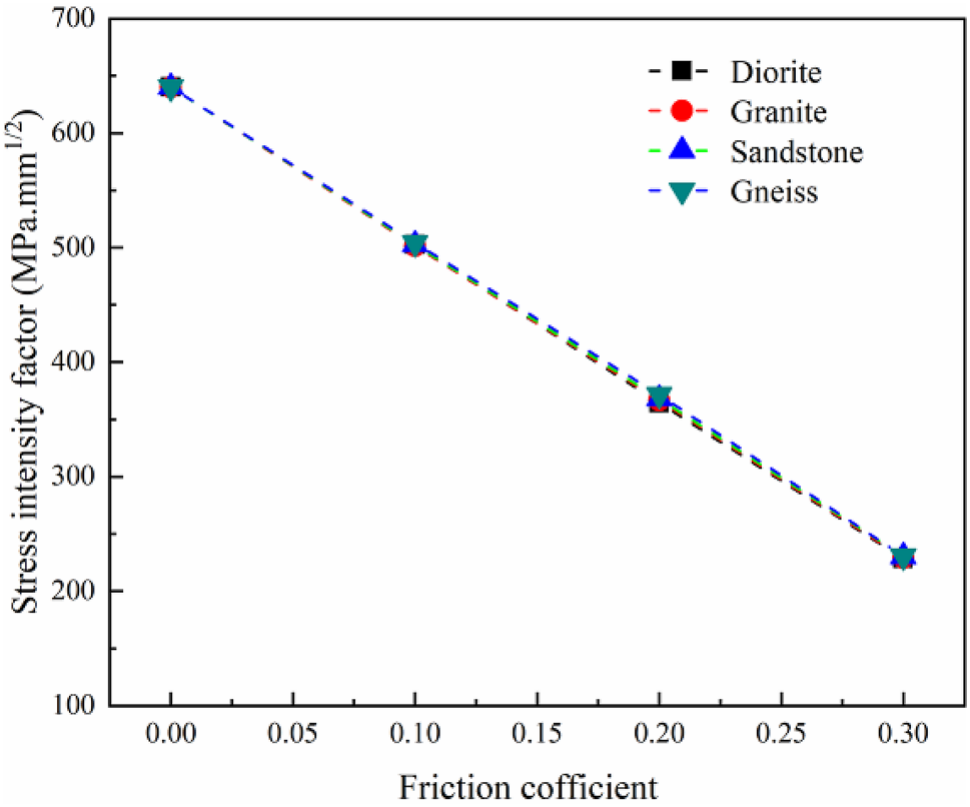
Variation of stress intensity factor with the elastic modulus of rock specimens under different friction coefficients.

As mentioned above, the friction coefficient of the three placement methods of the roller support for rock specimens with the NSCB configuration is within 0.3^27^, and the friction coefficient is controlled within this range in the later models and analysis. The load applied by the numerical model is 2000 N. In addition to the above material parameters and loads, other parameters input in the numerical model (such as specimen size and grid type) are the same as those of the numerical model of rock specimen with the NSCB configuration for model verification in Methods Section. It is worth noting that the effect of thickness or crack front length can affect the stress state as stated in some previous studies^51,52^. However, the variation law of stress intensity factor with friction coefficient is only focused in this study, and the two-dimensional analysis is only carried out. As shown in Fig. 4, the *K*_I_ decreases significantly with the increase of friction coefficient (as observed in Methods Section). At the same time, even if there is friction between the specimen surface and the roller support, the change of elastic modulus has little effect on the *K*_I_. This is independent of the small load applied in the model; in fact, the load applied in the model is much larger than the failure strength of the specimen. Rock specimens with the NSCB configuration are easily damaged under small load, and the crack opening displacement of the specimen with different material properties changes little, and the load applied at specimen is transformed into the vertical reaction force of the roller support on the specimen surface. In other words, the vertical reaction force of the roller support on the specimen surface rarely changes with the significant changes in material properties. Therefore, the change of rock material properties can be ignored when considering the influence of friction on the test results of *K*_Ic_ for rock specimens with the NSCB configuration.

### Effect of different load levels on the fracture toughness test

Generally, the friction force exerted by the roller support on the specimen is related to the friction coefficient and the vertical reaction force of the roller support on the specimen surface. The vertical reaction force of the roller support on the specimen surface is closely related to the load level applied to the specimen. In this study, four load levels are set (i.e. 500 N, 1000 N, 1500 N and 2000 N) to explore the effects of different load levels on the *K*_I_ of rock specimens with the NSCB configuration under the action of friction. The diorite is used in this test and the material parameters of the diorite in “Section Effect of elastic modulus of rock on the fracture toughness test” are selected to establish the model. The diorite is selected for several reasons: (1) the material properties are independent of the determination of *K*_I_; (2) diorite has a large elasticity modulus and small deformation in the model, the selection of diorite can avoid the difficult convergence of the model after the occurrence of large deformation. Other parameters input into the numerical model are the same as those of the numerical model of rock specimen with the NSCB configuration established in Methods Section.

**Figure 5:**
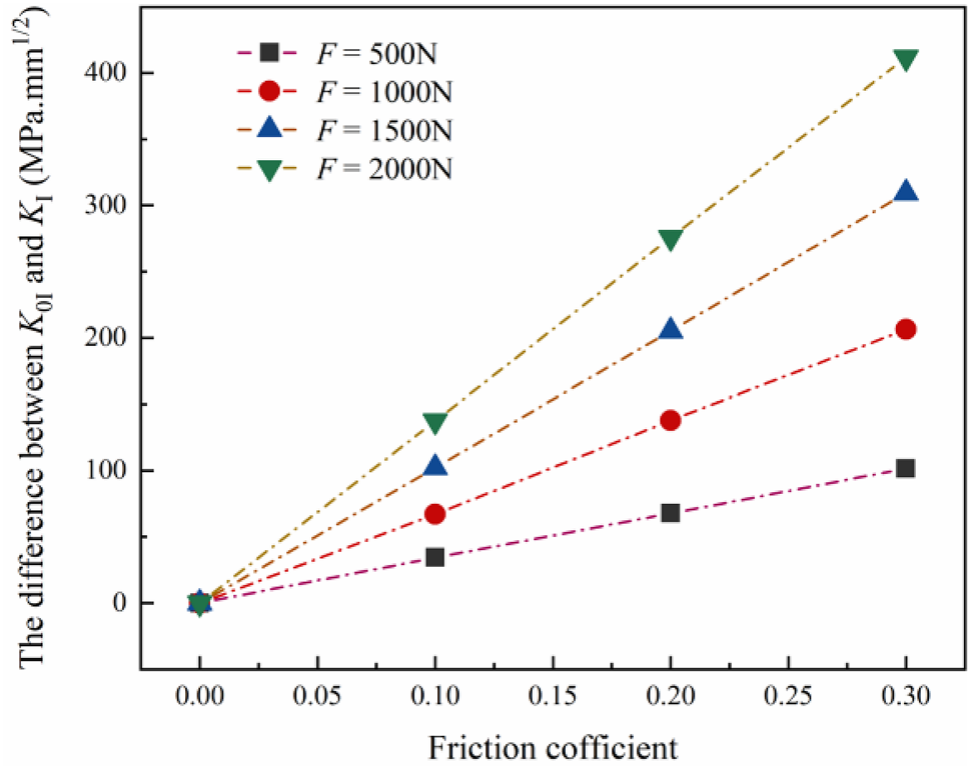
The decrease of stress intensity factor (the difference between the stress intensity factor in the friction-free condition and that in the friction condition) under different load levels varies with the friction coefficient.

Due to the friction between the roller support and the specimen surface, the crack opening is limited, and the *K*_I_ of the specimen is smaller than that of the specimen in the friction-free condition. As shown in Fig. 5, the *K*_I_ in friction-free condition (*K*_0I_) is taken as a reference, and the difference between the *K*_0I_ and the *K*_I_ (Δ*K*) under different load levels changes with the friction coefficient. At the same load level, the Δ*K* increases linearly with the increase of friction coefficient, which is consistent with the results in the previous study. At the same time, under the same friction coefficient, the Δ*K* increases with the increase of load level. This is mainly because the vertical reaction force exerted on the surface of the sample by roller support increases with the increase of load level, which leads to the increase of the friction force exerted by the roller support on the sample.

The *K*_I_ can be used to describe the stress field near the crack tip, which is the basis of linear elastic fracture mechanics (LEFM). Only for an ideal elastic material, when the *K*_I_ of the specimen is greater than the *K*_Ic_ of the material, the specimen fails immediately after the crack initiation. For elastic–plastic materials such as rock and concrete, plastic deformation is easy to occur near the crack tip, but the failure of the specimen is caused by the subcritical crack propagation after a period of time. Therefore, the stress state near the initial crack tip cannot result in the fracture of elastic–plastic materials directly. The modified maximum tangential stress fracture criterion proposed by Smith et al.^53^ can perfectly describe and predict the fracture problem of elastic–plastic materials. According to this criterion, when the tensile stress at the boundary of the fracture process zone is greater than the tensile strength of the material, the specimen will fracture. It should be noted that the elastic–plastic material has a large size of fracture process zone. Therefore, according to the maximum tangential stress fracture criterion, the stress distribution in the far-field at the front of the crack tip should be considered in the fracture analysis of elastic–plastic materials. Consequently, the variation of the stress field distribution at the front of the crack tip with the friction coefficient is also worth discussing.

**Figure 6:**
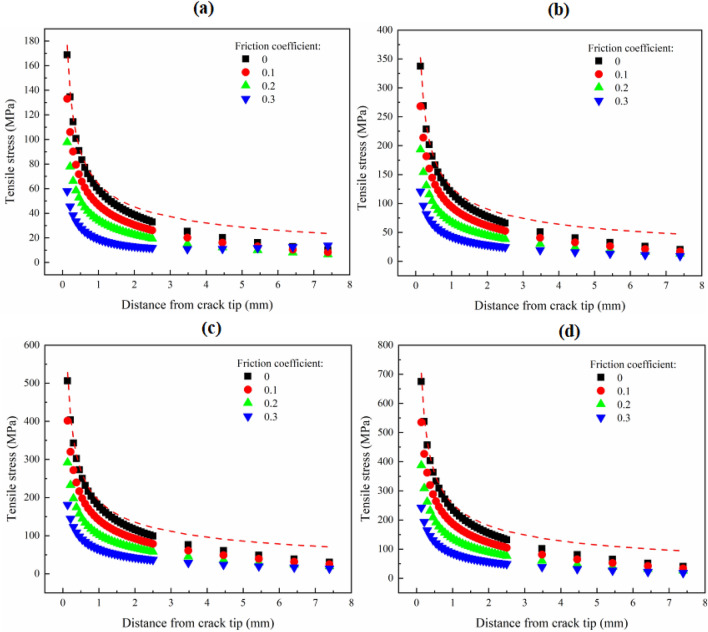
Variation of tensile stress at the front of crack tip with load level under different friction coefficients. Where (**a**) is load level of 500 N; (**b**) is load level of 1000 N; (**c**) is load level of 1500 N; and (**d**) is load level of 2000 N. Red dotted lines indicates the tensile stress determined by stress intensity factor that in the friction-free condition.

Figure 6 shows the changing trend of tensile stress at the front of the crack tip under different friction coefficients and load levels. At the same time, the tensile stress field determined by *K*_0I_ is also drawn in this figure. The tensile stress extracted from the numerical model established under the friction-free condition is almost equal to the tensile stress determined by *K*_0I_ in the region near the crack tip. However, with the increasing distance from the crack tip, the stress field distribution determined by *K*_0I_ gradually deviates from that extracted from the numerical model. At the same load level, with the increase of friction coefficient, the tensile stress at the front of the crack tip decreases gradually; with the increase of the load level, the decrease of the tensile stress at the front of the crack tip increases. In other words, according to the maximum tangential stress fracture criterion, a greater load on the specimen can be generated to the failure of the specimen in the fracture test under the condition of a large friction coefficient, rather than under the friction-free condition. At this time, if the *K*_Ic_ is calculated by the formula of *K*_I_ derived under the friction-free condition, the test value of *K*_Ic_ will be greater than the inherent fracture toughness of the material. Consequently, the difference between the tested *K*_Ic_ and the inherent *K*_Ic_ will be greater for the material with large fracture toughness. In addition, the tensile stress in the area far from the crack tip does not change significantly due to the change of the friction coefficient. This shows that the friction force between the roller support and the specimen surface has little effect on the high-order term coefficient of Williams expansion^39^.

### Effect of different spans of roller support on the fracture toughness test

The span of roller support is the key factor affecting the test results of *K*_Ic_ for rock specimens with the NSCB configuration. If the span of roller support is too small, mode I fracture hardly occurs in the specimen. When there is friction between the roller support and the specimen surface, the influence of the span of roller support on the test results of *K*_Ic_ becomes more complex. Therefore, four numerical models with different spans of roller support are established, that is, the ratio of the half span of roller support to the specimen radius is *S*/*R* = 0.2, 0.4, 0.6 and 0.8 respectively. To avoid the large deformation of the specimen, a load of 1000 N is applied to the model. Other parameters of the model are the same as those established in Methods Section.

**Figure 7:**
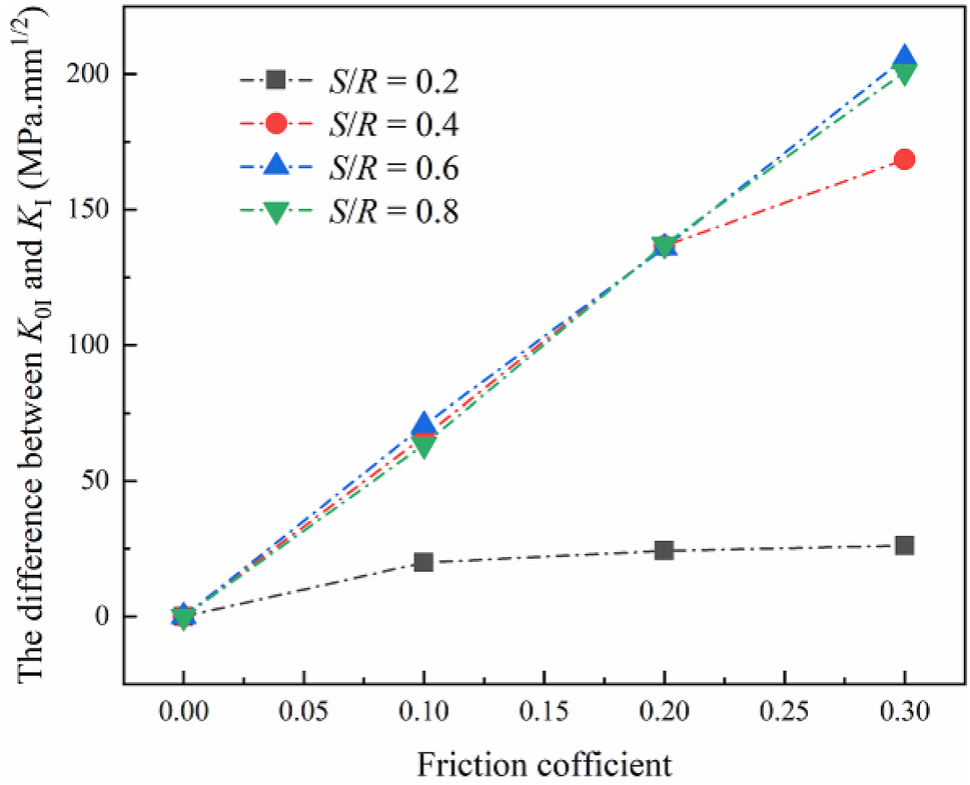
The decrease of stress intensity factor under different span of roller support varies with the friction coefficient.

As shown in Fig. 7, the Δ*K* increases with the increase of friction coefficient, which is consistent with the previous results. Compared with other spans of roller support, when *S*/*R* = 0.2, the change of *K*_I_ with friction coefficient is not significant. At the same time, under the same friction coefficient, the Δ*K* increases significantly with the increase of *S*/*R* from 0.2 to 0.4. However, when *S*/*R* changes from 0.4 to 0.8, the Δ*K* does not change. When the same load is applied to rock specimens with the NSCB configuration, the smaller the span of roller support, the smaller the angle between the bottom end face of the specimen and the vertical direction, the smaller the friction force acting on the specimen surface. Thus, when the span of roller support is small, the friction has little effect on the *K*_I_, and the Δ*K* hardly changes when *S*/*R* is 0.2. When *S*/*R* increases from 0.4 to 0.8, the angle between the bottom end face of the specimen and the vertical direction rarely changes, so the magnitude of the friction force is the same. When *S*/*R* increases from 0.2 to 0.4, the friction force changes greatly. As a result, the reduction of *K*_I_ shows different trends with the changes in the span of roller support.

**Figure 8:**
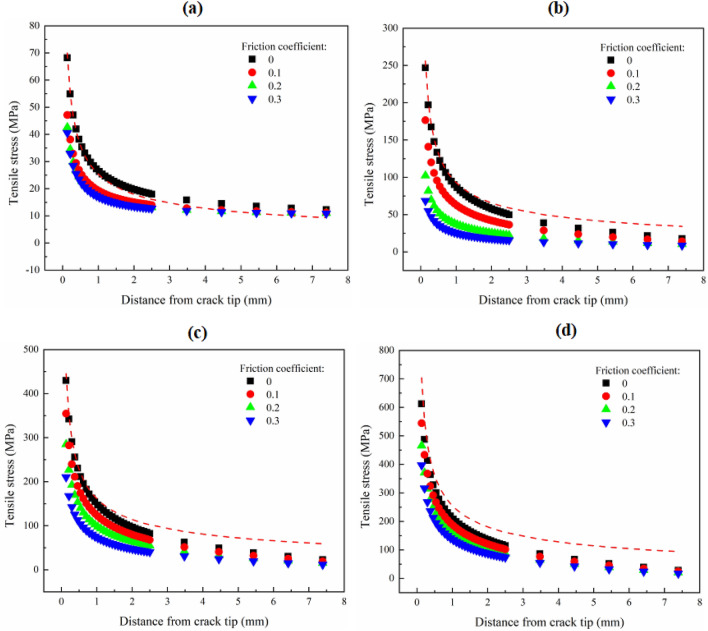
Variation of tensile stress at the front of crack tip with the span of roller support under different friction coefficients. Where (**a**) is *S*/*R* = 0.2; (**b**) is *S*/*R* = 0.4; (**c**) is *S*/*R* = 0.6; and (**d**) is *S*/*R* = 0.8. Red dotted lines indicates the tensile stress determined by stress intensity factor that in the friction-free condition.

Figure 8 shows the change of tensile stress at the front of the crack tip with friction coefficient under different spans of roller support. When *S*/*R* is 0.2, the tensile stress determined by *K*_0I_ is very close to that extracted from the numerical model. With the increase of the span of roller support, the difference between the two increases gradually. It indicates that the *K*_Ic_ tested under the condition of the small span of roller support can better reflect the inherent fracture characteristics of the material. With the increase of friction coefficient, the tensile stress at the front of the crack tip decreases gradually. Different from other spans of roller support, under the loading condition of *S*/*R* = 0.2, the tensile stress at the crack tip decreases significantly when the friction-free condition is transitioned to the friction condition, but does not change significantly with the increase of friction coefficient. Under different spans of roller support, the tensile stress in the area far from the crack tip is less affected by the friction coefficient. This further shows that the friction force has little effect on the coefficients of the higher-order terms of the Williams expansion.

The span of roller support of the NSCB configuration recommended by ISRM for the fracture toughness test is from *S*/*R* = 0.5 to *S*/*R* = 0.8^4^. It can be seen from Fig. 7 that within this span of roller support, the influence of friction on the stress intensity factor is the same at the same load level. According to the theory of linear elastic fracture mechanics, for the loading condition with a large span of roller support, the rock will fracture at a small load level, and the influence of friction on the stress intensity factor will be relatively small. Therefore, a larger span should be selected to carry out the experiment. However, considering the difference between the real tensile stress distribution at the crack tip front of the specimen and that determined by the stress intensity factor, it can be found that the smaller the span, the smaller the difference. Based on the above two aspects, for rock materials with high strength, the large span of roller support should be selected due to the small fracture process zone length; for rock materials with low strength, the small span of roller support should be selected according to the actual loading conditions.

### Effect of different friction coefficients on size effect of fracture toughness

The size effect of fracture toughness in rock materials has always been emphasized in rock fracture mechanics^54–56^. Because rock specimens with the NSCB configuration are convenient in sample processing and loading test, and the transformation from mode I loading to mode II loading conditions can be realized by adjusting the position of the roller support, the NSCB configuration has been widely used in the study of size effect of the fracture toughness of mode I and mode II in rock. However, the effect of friction on the fracture toughness test in rock specimens with the NSCB configuration is rarely considered^29–31^. From the analysis of the previous sections, it can be found that friction has a great influence on the fracture toughness test. For the same material, the *K*_Ic_ value can differ several times under different experimental conditions of friction coefficient. At present, many studies have tried to establish the mathematical relationship between *K*_Ic_ and the specimen size of the rock^29–31,57–60^. However, it is not clear how the existence of friction will affect the establishment of the mathematical relationship between them. Therefore, numerical models with different specimen sizes and different friction coefficients are established in this study.

**Figure 9:**
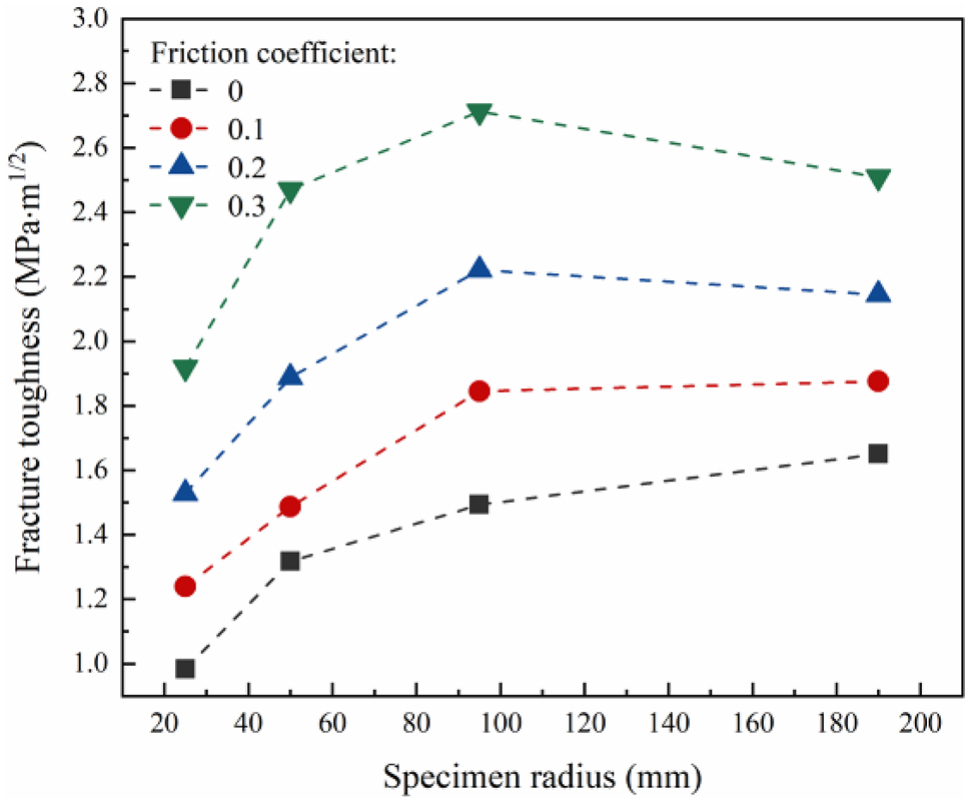
Variation of fracture toughness with specimen size under different friction coefficients.

To more simulate the real fracture situation of rock, the mode I fracture test data of marble specimens with NSCB configurations in different sizes (available in the literature^57^) are employed to establish the numerical model. Table 3 shows the detailed test data. It is worth noting that the test data are assumed to be obtained under friction-free loading conditions in this study. In the presence of friction, the load is applied on the numerical model until the *K*_I_ is equal to the tested *K*_Ic_ in Table 3, and the load at this time is substituted into the calculation formula of *K*_I_ deduced under the friction-free condition. Then the calculated *K*_I_ is the *K*_Ic_ under the corresponding friction coefficient. According to the above steps, the variation law of *K*_Ic_ with specimen size under different friction coefficients is obtained. As shown in Fig. 9, under the friction-free condition, the *K*_Ic_ increases with the increase of specimen size, and the increasing trend is gradually reduced. However, when the specimen size increases from 95 to 190 mm, the *K*_Ic_ decreases at the friction coefficient of 0.2 and 0.3. It indicates that when the specimen size increases to a certain extent, the influence of the friction on the *K*_Ic_ decreases.

**Table 3:** The test data of fracture toughness of rock specimens with NSCB configuration in different sizes from Ayatollahi et al.^38^.

Geometry	Specimen dimensions (*R* × *a* × *t*)	*K*_Ic_ (MPa·m^1/2^)
NSCB (*a*/*R* = 0.5, *S*/*R* = 0.5)	25 × 12.5 × 1 (mm × mm)	0.983
	50 × 25 × 1 (mm × mm)	1.317
	95 × 47.5 × 1 (mm × mm)	1.493
	190 × 95 × 1 (mm × mm)	1.65

Many studies have reported the size effect of *K*_Ic_ in rock. To effectively apply the *K*_Ic_ tested in the laboratory to the engineering site, the relationship between the *K*_Ic_ and the specimen size must be established. The size effect law proposed by Bazant et al.^61,62^ has been widely used to predict the *K*_Ic_ if the specimen size tends to infinity. In the next part, the impact of different friction coefficients on the establishment of Bazant’s size effect law is discussed.

#### Description of Bazant's size effect law

According to the LEFM, when a structure is subjected to nominal stress *σ*_N_, the expression of *K*_I_ is as follows:

1 $$K_{{\text{I}}} = \sigma_{{\text{N}}} \sqrt L k\left( \alpha \right)$$

where *L* is the characteristic length (equal to the radius of the specimen with NSCB configuration), and *k*(*α*) denotes dimensionless functions. The nominal stress *σ*_N_ of the specimen with NSCB configuration can be defined by:

$$\sigma_{{\text{N}}} = \frac{P}{tR}$$

where *P* is the fracture load, and *t* and *R* are the thickness and radius of the specimen with the NSCB configuration, respectively. Therefore, the energy release rate *G*(*α*) per unit length of crack propagation can be written as:

2 $$G\left( \alpha \right) = \frac{{K_{{\text{I}}}^{{2}} }}{E} = \frac{{\sigma_{{\text{N}}}^{{2}} L}}{E}g\left( \alpha \right)$$

Here *g*(*α*) = *k*(*α*)^2^ represents the dimensionless energy release rate. Based on the equivalent LEFM, when the equivalent crack tip is at a certain distance *c* ahead of the initial crack tip, the maximum loads of specimens with various sizes can be reached. Therefore, the length of *c* should also be considered in the effective crack length in the calculation of the energy release rate. When the specimen size tends to infinity, the corresponding value of *c* in size effect law is denoted as *c*_f_*.*, and the following equation can be established:

3 $$G\left( {\alpha_{0} + c_{{\text{f}}} /L} \right) = \frac{{\sigma_{{{\text{Nu}}}}^{{2}} L}}{E}g\left( {\alpha_{0} + c_{{\text{f}}} /L} \right) = G_{{\text{f}}}$$

where *G*_f_ is the fracture energy and belongs to the material constant; *σ*_Nu_ is the nominal failure stress of the specimen and *α*_0_ is the ratio of the length of the prefabricated crack to the characteristic length of the specimen. By approximating *g*(*α*_0_ + *c*_f_/*L*) with its Taylor series expansion at *α*_0_, the linear term of the expansion is only retained, Eq. (4) can be obtained:

4 $$\sigma_{{{\text{Nu}}}} = \sqrt {\frac{{E*G_{{\text{f}}} }}{{Lg\left( {\alpha_{0} } \right) + c_{{\text{f}}} g^{^{\prime}} \left( {\alpha_{0} } \right)}}}$$

Equation (4) is replaced with the following simple variables as follows:

5 $$X = L,\quad Y = \sigma_{{\text{N}}}^{{ - 2}}$$

6 $$A = \frac{{g\left( {\alpha_{0} } \right)}}{{E * G_{{\text{f}}} }};\quad C = \frac{{c_{{\text{f}}} g^{^{\prime}} \left( {\alpha_{0} } \right)}}{{E * G_{{\text{f}}} }}$$

Then the relationship between failure stress and specimen size can be simplified as follows:

7 $$Y = AX + C$$

where *A* and *C* are constants related to *E*, *G*_f_ and *c*_f_. The values of *A* and *C* can be obtained by the linear fitting method with sufficient test data. According to the relationship between failure stress and specimen size established based on Bazant’s size effect law, the *K*_Ic_ of specimens in any size can be predicted.

#### Establishment of Bazant’s size effect law under various friction coefficients

**Figure 10:**
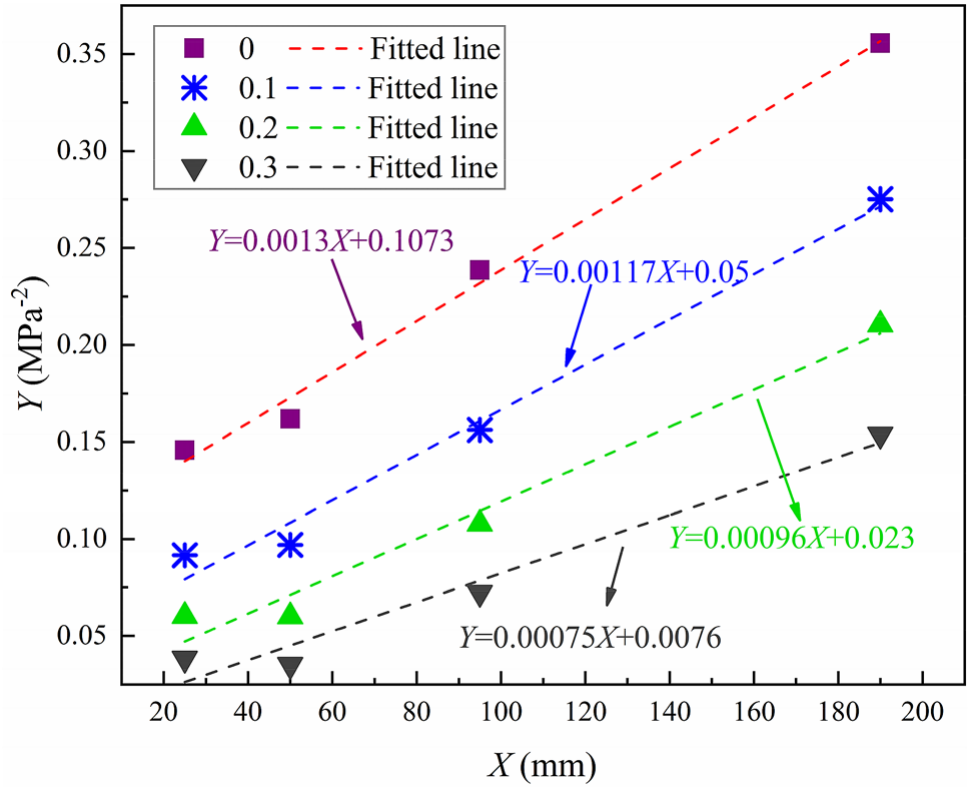
Failure loads transformed under different friction coefficients with the change of specimen size.

The failure load of the specimen under each friction coefficient can be obtained from the numerical model and substituted into Eq. (5) to calculate the values of *X* and *Y*. Then the values of *X* and *Y* are drawn in Fig. 10. It can be seen that whether there is friction or not, the variation law of *K*_Ic_ with specimen size obtained from the test conforms to Bazant’s size effect law. However, it is difficult to distinguish whether the fracture test is affected by the friction from the fitting goodness of the data. In this study, the linear fitting on the test data of each group is performed to obtain the slope *A* and intercept *C* of the straight line, as shown in Table 4. According to Eq. (6), the fracture energy and the fracture process zone length can be obtained when the specimen size tends to infinity; however, the changes of the fracture energy and the fracture process zone length with the friction coefficient are only discussed in this study. As described in Eq. (6), the fracture energy is only related to the value of *A*, and the two are inversely proportional. The fracture process zone length is only proportional to the ratio of *C* and *A*. Therefore, the values of *A* and *C* under different friction coefficients are compared to obtain the relationship among the fracture energy and the fracture process zone length and the friction coefficient. It can be seen that with the increase of friction coefficient, the value of *A* gradually decreases, indicating that the fracture energy gradually increases. The friction coefficient increases from 0 to 0.3, and the fracture energy increases by 42%. At the same time, with the increase of friction coefficient, the ratio of *C* to *A* gradually decreases, indicating that the fracture process zone length is gradually decreased. When the friction coefficient increases from 0 to 0.3, the fracture process zone length decreases by about 90%. In summary, although the existence of friction does not affect the establishment of Bazant's size effect law, it has a significant impact on the prediction of the fracture energy and the fracture process zone length when the specimen size tends to infinity.

**Table 4:** The *A* and *C* values under different friction coefficients.

Friction coefficient	*A*	*C*	*C*/*A*	Coefficient of determination
0	0.0013	0.1073	82.538	0.99
0.1	0.00117	0.05	42.735	0.985
0.2	0.00096	0.023	23.958	0.976
0.3	0.00075	0.0076	10.133	0.967

**Figure 11:**
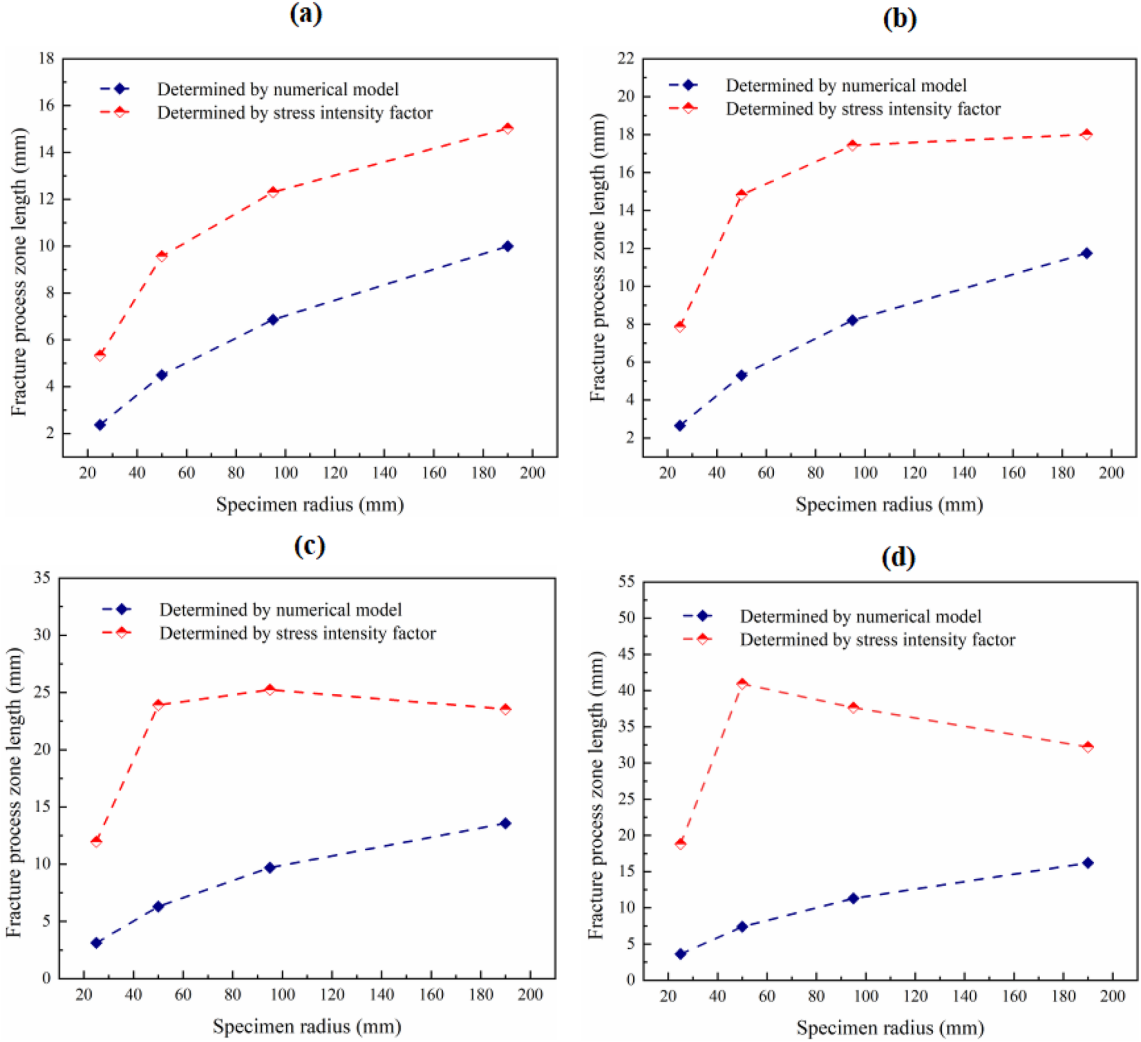
Variation of the fracture process zone length with specimen size under different friction coefficients. Where (**a**) is friction coefficient of 0; (**b**) is friction coefficient of 0.1; (**c**) is friction coefficient of 0.2; and (**d**) is friction coefficient of 0.3.

In addition to the prediction of fracture energy when the specimen size tends to infinity, the accurate determination of the fracture process zone length of various specimen sizes is also very important for the prediction of failure load of specimen^14–16,57–59^. Many studies believe that the boundary of the fracture process zone is the range where the tensile stress of the crack tip is greater than or equal to the tensile strength of the material^16,57,63–65^. As mentioned earlier, there are two methods to determine the tensile stress at the front of the crack tip. In the first method, the tensile stress at the front of the crack tip is directly determined by the *K*_I_ alone; in the second method, the higher-order term of Williams expansion is considered, the tensile stress in the far-field of the crack tip can be described accurately. Obviously, the fracture process zone length calculated by the two methods of determining the tensile stress is different. The tensile strength of marble used for the fracture test is 5.37 MPa, then the fracture process zone length of the specimen under each friction coefficient can be obtained. As shown in Fig. 11, the fracture process zone length determined by the *K*_I_ is much larger than that determined by the numerical model. According to the determination criteria of the fracture process zone mentioned earlier, the fracture process zone length will be large because the tensile strength of marble in this test is small. However, the *K*_I_ can only accurately describe the stress field near the crack tip. Therefore, applying the *K*_I_ to determine the fracture process zone length will cause a great error. In addition, with the increase of friction coefficient, the fracture process zone length also increases. This is different from the previous results in that the fracture process zone length of the specimen with an infinite size decreases with the increase of the friction coefficient. This shows that the estimation error of fracture process zone length increases with the increase of the friction coefficient. In addition, with the increase of specimen size, the effect of friction coefficient on the fracture process zone length increases gradually. It indicates that for small-size specimens, the effect of friction on the estimation of fracture process zone length is small.

## Conclusions

In this study, two test methods for determining the fracture toughness in rock specimens with NSCB configuration and CSTBD configuration are considered. The influence of contact friction in the testing process on the test results of fracture toughness is analyzed in detail, and the following conclusions are drawn as follows: (1) The test results of rock specimens with CSTBD configuration used in the fracture toughness test are less affected by the friction between the rigid plate and specimen surface. For rock specimens with NSCB configuration, the *K*_I_ of the specimen decreases with the increase of friction coefficient under the same load, indicating that the failure load of the specimen increases with the increase of friction coefficient. If the influence of the friction is ignored, the tested *K*_Ic_ deviates significantly from the inherent *K*_Ic_ of rock with the increase of friction coefficient; (2) For rock specimens with the NSCB configuration, the change of material elastic modulus under the same load level rarely affects the influence of friction on the *K*_I_ of the specimen. With the increase of load level, the weakening effect of friction on the *K*_I_ of the specimen increases. For the same friction coefficient, the weakening effect of friction on the *K*_I_ under the loading condition of the small span of roller support is much smaller than that under the loading condition of the large span of roller support. At the same time, the weakening effect of friction on *K*_I_ is no longer enhanced when the span of roller support increases to a certain extent; and (3) For the size effect of fracture toughness in rock, the *K*_Ic_ obtained under different friction coefficients increases with the increase of specimen size. At the same time, the relationship between specimen failure load and specimen size under different friction coefficients can be well described by Bazant’s size effect law. As the friction coefficient increases, the estimated value of the fracture energy increases, while the fracture process zone length decreases for the specimen with an infinite size. When the specimen size is at the laboratory scale, the fracture process zone length gradually increases with the increase of the friction coefficient.

## References

[1] Franklin J (1988). Suggested methods for determining the fracture toughness of rock. Int. J. Rock Mech. Min. Sci..

[2] Aliha MRM, Ayatollahi MR (2014). Rock fracture toughness study using cracked chevron notched Brazilian disc specimen under pure modes I and II loading – A statistical approach. Theoret. Appl. Fract. Mech..

[3] Fowell, R., Hudson, J., Xu, C. & Zhao, X. in *International Journal of Rock Mechanics and Mining Sciences and Geomechanics Abstracts.* 322A.

[4] Kuruppu MD, Obara Y, Ayatollahi MR, Chong KP, Funatsu T (2014). ISRM-suggested method for determining the mode I static fracture toughness using semi-circular bend specimen. Rock Mech. Rock Eng..

[5] Tutluoglu L, Keles C (2011). Mode I fracture toughness determination with straight notched disk bending method. Int. J. Rock Mech. Min. Sci..

[6] Aliha MRM, Bahmani A, Akhondi S (2015). Determination of mode III fracture toughness for different materials using a new designed test configuration. Mater. Des..

[7] Ayatollahi MR, Mahdavi E, Alborzi MJ, Obara Y (2016). Stress intensity factors of semi-circular bend specimens with straight-through and chevron notches. Rock Mech. Rock Eng..

[8] Mahdavi E, Aliha MRM, Bahrami B, Ayatollahi MR (2020). Comprehensive data for stress intensity factor and critical crack length in chevron notched semi-circular bend specimen subjected to tensile type fracture mode. Theoret. Appl. Fract. Mech..

[9] Yamauchi Y, Nakano M, Kishida K, Okabe T (2000). Measurement of fracture toughness for brittle materials under mixed-mode impact loading using center-notched disk specimen. J. Soc. Mater. Sci. Jpn..

[10] Zhou J, Wang Y, Xia Y (2006). Mode-I fracture toughness of PMMA at high loading rates. J. Mater. Sci..

[11] Xie Q, Li S-X, Liu X-L, Gong F-Q, Li X-B (2020). Effect of loading rate on fracture behaviors of shale under mode I loading. J. Central South Univ..

[12] Aliha MRM, Mahdavi E, Ayatollahi MR (2017). The influence of specimen type on tensile fracture toughness of rock materials. Pure Appl. Geophys..

[13] Wei MD, Dai F, Xu N-W, Liu Y, Zhao T (2018). A novel chevron notched short rod bend method for measuring the mode I fracture toughness of rocks. Eng. Fract. Mech..

[14] Wei MD, Dai F, Zhou J-W, Liu Y, Luo J (2018). A further improved maximum tangential stress criterion for assessing mode I fracture of rocks considering non-singular stress terms of the Williams expansion. Rock Mech. Rock Eng..

[15] Aliha MRM, Mahdavi E, Ayatollahi MR (2018). Statistical analysis of rock fracture toughness data obtained from different Chevron notched and straight cracked mode I specimens. Rock Mech. Rock Eng..

[16] Aliha MRM, Sistaninia M, Smith DJ, Pavier MJ, Ayatollahi MR (2012). Geometry effects and statistical analysis of mode I fracture in guiting limestone. Int. J. Rock Mech. Min. Sci..

[17] Mirsayar MM, Razmi A, Aliha MRM, Berto F (2018). EMTSN criterion for evaluating mixed mode I/II crack propagation in rock materials. Eng. Fract. Mech..

[18] Bahmani A (2021). On the comparison of two mixed-mode I + III fracture test specimens. Eng. Fract. Mech..

[19] Aliha MRM, Bahmani A (2017). Rock fracture toughness study under mixed mode I/III loading. Rock Mech. Rock Eng..

[20] He J, Liu L, Yang H, Aliha MRM, Karimi HR (2021). Contribution of interface fracture mechanism on fracture propagation trajectory of heterogeneous asphalt composites. Appl. Sci..

[21] Saed SA (2022). Full range I/II fracture behavior of asphalt mixtures containing RAP and rejuvenating agent using two different 3-point bend type configurations. Constr. Build. Mater..

[22] Yang D, Karimi HR, Aliha MRM (2021). Comparison of testing method effects on cracking resistance of asphalt concrete mixtures. Appl. Sci..

[23] Aliha MRM, Karimi HR, Abedi M (2022). The role of mix design and short glass fiber content on mode-I cracking characteristics of polymer concrete. Constr. Build. Mater..

[24] Aliha MRM, Razmi A, Mousavi A (2018). Fracture study of concrete composites with synthetic fibers additive under modes I and III using ENDB specimen. Constr. Build. Mater..

[25] Dehghany M, Saeidi Googarchin H, Aliha MRM (2017). The role of first non-singular stress terms in mixed mode brittle fracture of V-notched components: An experimental study. Fatigue Fract. Eng. Mater. Struct..

[26] Wei MD, Dai F, Xu N-W, Zhao T, Liu Y (2017). An experimental and theoretical assessment of semi-circular bend specimens with chevron and straight-through notches for mode I fracture toughness testing of rocks. Int. J. Rock Mech. Min. Sci..

[27] Sedighi I, Ayatollahi MR, Bahrami B (2020). A statistical approach on the support type effect on mode I fracture toughness determined using semi-circular bend (SCB) specimen. Eng. Fract. Mech..

[28] Wu Y, Yin T, Tan X, Zhuang D (2021). Determination of the mixed mode I/II fracture characteristics of heat-treated granite specimens based on the extended finite element method. Eng. Fract. Mech..

[29] Lu DX, Bui HH, Saleh M (2021). Effects of specimen size and loading conditions on the fracture behaviour of asphalt concretes in the SCB test. Eng. Fract. Mech..

[30] Ghouli S, Bahrami B, Ayatollahi MR, Driesner T, Nejati M (2021). Introduction of a scaling factor for fracture toughness measurement of rocks using the semi-circular bend test. Rock Mech. Rock Eng..

[31] Xu S, Malik MA, Li Q, Wu Y (2016). Determination of double-K fracture parameters using semi-circular bend test specimens. Eng. Fract. Mech..

[32] Li Y, Dai F, Wei M, Du H (2020). Numerical investigation on dynamic fracture behavior of cracked rocks under mixed mode I/II loading. Eng. Fract. Mech..

[33] Wei MD, Dai F, Xu N-W, Liu Y, Zhao T (2017). Fracture prediction of rocks under mode I and mode II loading using the generalized maximum tangential strain criterion. Eng. Fract. Mech..

[34] Chen X, Tang J, Zhang N, Dai F, Wei M (2022). Fracture analysis of three-point bending notched granite beams under prepeak and postpeak cyclic loading by digital image correlation and acoustic emission techniques. Fatigue Fract. Eng. Mater. Struct..

[35] Aliha MRM, Jafari Haghighatpour P, Tavana A (2022). Application of asymmetric semi-circular bend test for determining mixed mode I + II fracture toughness of compacted soil material. Eng. Fract. Mech..

[36] Fuan S, Ke M, Kanghe L, Kun L, Aliha MRM (2021). Influence of specimen geometry on mode I fracture toughness of asphalt concrete. Constr. Build. Mater..

[37] Shahryari N, Keymanesh MR, Aliha MRM (2021). Specimen type effect on measured low-temperature fracture toughness of asphalt concrete. Fatigue Fract. Eng. Mater. Struct..

[38] Pakdaman AM, Moosavi M, Mohammadi S (2019). Experimental and numerical investigation into the methods of determination of mode I static fracture toughness of rocks. Theoret. Appl. Fract. Mech..

[39] Williams ML (1957). On the stress distribution at the base of a stationary crack. J. Appl. Mech..

[40] Wei M, Dai F, Liu Y, Li A, Yan Z (2021). Influences of loading method and notch type on rock fracture toughness measurements: From the perspectives of T-stress and fracture process zone. Rock Mech. Rock Eng..

[41] Wei MD, Dai F, Liu Y, Xu N-W, Zhao T (2018). An experimental and theoretical comparison of CCNBD and CCNSCB specimens for determining mode I fracture toughness of rocks. Fatig. Fract. Eng. Mater. Struct..

[42] Aliha MRM, Ayatollahi MR, Akbardoost J (2012). Typical upper bound-lower bound mixed mode fracture resistance envelopes for rock material. Rock Mech. Rock Eng..

[43] Wang H (2022). A complex analysis of stress intensity factors for two asymmetric and unequal collinear cracks in rocks subjected to compressive and shear loads. Theoret. Appl. Fract. Mech..

[44] Dong S, Wang Y, Xia Y (2004). Stress intensity factors for central cracked circular disk subjected to compression. Eng. Fract. Mech..

[45] Mustafa M, Amroune S, Tahiri A, Hachi B (2020). Brittle fracture investigation from disc specimen weakened by U- notch in mixed mode I + II. Eng. Solid Mech..

[46] Mohammad Aliha MR, Ghesmati Kucheki H, Asadi MM (2021). On the use of different diametral compression cracked disc shape specimens for introducing mode III deformation. Fatig. Fract. Eng. Mater. Struct..

[47] Bahmani A, Nemati S (2021). Fracture resistance of railway ballast rock under tensile and tear loads. Eng. Solid Mech..

[48] Fayed A (2018). Numerical evaluation of mode I/II SIF of quasi-brittle materials using cracked semi-circular bend specimen. Eng. Solid Mech..

[49] Mirsayar M, Shi X, Zollinger D (2017). Evaluation of interfacial bond strength between Portland cement concrete and asphalt concrete layers using bi-material SCB test specimen. Eng. Solid Mech..

[50] Aliha MRM, Pakzad R, Ayatollahi MR (2014). Numerical analyses of a cracked straight-through flattened Brazilian disk specimen under mixed-mode loading. J. Eng. Mech..

[51] Aliha MRM, Saghafi H (2013). The effects of thickness and Poisson’s ratio on 3D mixed-mode fracture. Eng. Fract. Mech..

[52] Imani DM, Aliha MRM, Linul E, Marsavina L (2022). A suitable mixed mode I/II test specimen for fracture toughness study of polyurethane foam with different cell densities. Theoret. Appl. Fract. Mech..

[53] Smith D, Ayatollahi MR, Pavier MJ (2008). The role of T-stress in brittle fracture for linear elastic materials under mixed mode loading. Fatig. Fract. Eng. Mater. Struct..

[54] Bidadi J, Akbardoost J, Aliha MRM (2020). Thickness effect on the mode III fracture resistance and fracture path of rock using ENDB specimens. Fatig. Fract. Eng. Mater. Struct..

[55] Akbardoost J, Ayatollahi MR, Aliha MRM, Pavier MJ, Smith DJ (2014). Size-dependent fracture behavior of Guiting limestone under mixed mode loading. Int. J. Rock Mech. Min. Sci..

[56] Aliha MRM, Ayatollahi MR, Smith DJ, Pavier MJ (2010). Geometry and size effects on fracture trajectory in a limestone rock under mixed mode loading. Eng. Fract. Mech..

[57] Ayatollahi MR, Akbardoost J (2013). Size effects in mode II brittle fracture of rocks. Eng. Fract. Mech..

[58] Sangsefidi M, Akbardoost J, Zhaleh AR (2021). Assessment of mode I fracture of rock-type sharp V-notched samples considering the size effect. Theoret. Appl. Fract. Mech..

[59] Ayatollahi MR, Akbardoost J (2012). Size effects on fracture toughness of quasi-brittle materials – A new approach. Eng. Fract. Mech..

[60] Saouma V, Natekar D, Hansen E (2003). Cohesive stresses and size effects in elasto-plastic and quasi-brittle materials. Int. J. Fract..

[61] Bažant ZP, Kim JK, Pfeiffer PA (1986). Nonlinear fracture properties from size effect tests. J. Struct. Eng..

[62] Bažant, Z.P. & Planas, J. *Fracture and size effect in concrete and other quasibrittle materials*. (Routledge, 1997).

[63] Fan Y, Zhu Z, Zhao Y, Zhou C, Zhang X (2019). The effects of some parameters on perforation tip initiation pressures in hydraulic fracturing. J. Petrol. Sci. Eng..

[64] Wang C, Zhu ZM, Liu HJ (2016). On the I-II mixed mode fracture of granite using four-point bend specimen. Fatig. Fract. Eng. Mater. Struct..

[65] Ayatollahi MR, Sistaninia M (2011). Mode II fracture study of rocks using Brazilian disk specimens. Int. J. Rock Mech. Min. Sci..

